# *O*-Mannosyltransferase CfPmt4 Regulates the Growth, Development and Pathogenicity of *Colletotrichum fructicola*

**DOI:** 10.3390/jof10050330

**Published:** 2024-05-01

**Authors:** Di Yang, Lan Luo, Yadi Liu, He Li

**Affiliations:** 1 Key Laboratory of National Forestry and Grassland Administration on Control of Artificial Forest Diseases and Pests in South China, Central South University of Forestry and Technology, Changsha 410004, China; yd502538975@163.com (D.Y.); 20200230@csuft.edu.cn (L.L.); 2 Green Home Engineering Technology Research Center in Hunan, Central South University of Forestry and Technology, Changsha 410004, China; t20060695@csuft.edu.cn

**Keywords:** *O*-mannosyltransferase, appressorium formation, stress response, pathogenicity, *Colletotrichum fructicola*

## Abstract

*Camellia oleifera* is a woody, edible-oil plant native to China. Anthracnose is the major disease of *Ca. oleifera*, and *Colletotrichum fructicola* is the main epidemic pathogen. Our previous research indicated that CfHac1 (homologous to ATF/CREB1) and CfGcn5 (general control nonderepressible 5, Gcn5) are integral to key cellular processes that govern fungal development and pathogenesis. Further transcriptomic analyses of the CfHac1 and CfGcn5 mutants, particularly under conditions of endoplasmic reticulum (ER) stress, hold the potential to unveil additional genes implicated in this critical cellular response. We identified all *OST/PMT* (oligosaccharyltransferase/Protein *O*-Mannosyltransferases) genes in *C. fructicola* and analyzed their expression levels. To elucidate novel glycosylation-related genes that may be important for the virulence of *C. fructicola*, we took an unbiased transcriptomic approach comparing wild-type and the ∆*Cfhac1* mutant. Notably, all *OST/PMT* genes were induced by dithiothreitol and down-regulated in the Δ*Cfhac1* mutant, yet only the *CfPMT4* (Protein *O*-Mannosyltransferases 4) gene (A04626) was unaffected in the Δ*Cfgcn5*. The results of targeted gene deletion experiments indicate that *CfPMT4* plays a crucial role in both vegetative growth and conidiation. Additionally, our investigation revealed that the Δ*Cfpmt4* exhibits deficiencies in appressorium formation, as well as in its response to cell wall integrity and endoplasmic reticulum stresses. Furthermore, the mutant displayed impaired glycogen metabolism, which may contribute to reduced penetration ability. Overall, CfPmt4, an *O*-mannosyltransferase, controls the growth, development, and pathogenicity of *Colletotrichum fructicola*. Understanding the function of the *CfPMT4* homolog could provide a potential molecular target for controlling *Ca. oleifera* anthracnose.

## 1. Introduction

Tea-oil (*Camellia oleifera*) is an important woody edible oil plant in China [1]. *Colletotrichum* species cause anthracnose, a major disease of *Ca. oleifera*. We have recently discovered that the pathogens responsible for anthracnose in *Ca. oleifera* include *Colletotrichum fructicola*, *C. gloeosporioides*, *C. siamense*, and *C. camelliae*. Among these, *C. fructicola* was identified as the primary epidemic pathogen [2,3,4].

The bZIP transcription factor Cfhac1 and the histone acetyltransferase CfGcn5 serve as pivotal molecular regulators in the life cycle and pathogenicity of *C. fructicola* [5,6,7]. This pathogen inflicts considerable economic damage on annual tea-oil production. Cfhac1, a leucine zipper transcription factor, shares significant homology with Hac1 in other fungal species. The targeted deletion of Cfhac1 leads to a cascade of physiological impairments, including diminished vegetative growth, conidiation, appressorium formation, osmotic stress tolerance, and overall pathogenicity. Transcriptomic analyses further illuminate Cfhac1’s role in modulating genes associated with the unfolded protein response and endoplasmic reticulum (ER) stress. These findings underscore Cfhac1’s indispensable function in orchestrating fungal development and virulence, primarily through mediating ER stress responses in *C. fructicola*. Conversely, CfGcn5 encodes a nuclear-localized histone acetyltransferase. Its targeted deletion results in compromised growth, conidiation, stress responses, appressorium formation, and pathogenicity. Domain deletion studies reveal that the NLS region, along with the HAT and Bromo domains, are crucial for CfGcn5’s nuclear localization and functional roles. In summary, both Cfhac1 and CfGcn5 are integral to key cellular processes that govern fungal development and pathogenesis. However, Cfhac1 emerges as a more central regulator, particularly through its involvement in ER stress responses, which are critical for fungal virulence. Understanding the roles and interplay of Cfhac1 and CfGcn5 offers valuable insights into the molecular mechanisms that regulate anthracnose disease development.

Further transcriptomic analyses of the Cfhac1 and CfGcn5 mutants, particularly under conditions of ER stress, hold the potential to unveil additional genes implicated in this critical cellular response [6,8]. The functional elucidation of these newly identified genes will furnish a more comprehensive understanding of the mechanisms by which *C. fructicola* navigates host-induced ER stress during the infection process. This enhanced insight could be instrumental in developing targeted interventions for this economically and ecologically significant pathogen.

## 2. Materials and Methods

### 2.1. Strains and Culture Conditions

The *C. fructicola* wild-type strain CFLH16 was isolated and archived by our laboratory [2], while the *Saccharomyces cerevisiae* strain XK-125 was preserved within our laboratory. The mutant Δ*Cfpmt4* and the complementary strain Δ*Cfpmt4*/*CfPMT4* were obtained by this experiment. All of the strains were cultured on complete medium (CM) plate at 28 °C in darkness unless the medium is stated.

### 2.2. RNA-seq Sample Preparation and Data Analysis

The *C. fructicola* wild-type strain CFLH16, Δ*CfGcn5* (general control nonderepressible 5, Gcn5) and Δ*CfHac1* (homologous to ATF/CREB1) were cultivated on potato dextrose agar (PDA) for a duration of four days. Post cultivation, samples of hyphae were excised from the colony edges and subsequently transferred into a 100 mL aliquot of Czapek–Dox liquid medium. The inoculated medium was then incubated at a constant temperature of 28 °C with a rotational speed of 60 rpm for a time span of 48 h. After the incubation period, the culture was filtered through three layers of sterilized lens paper, separating the hyphae and spores from the liquid medium, and the filtrate was collected in a 50 mL centrifuge tube. The filtrate was subsequently centrifuged at 5000 rpm for three minutes. The supernatant was discarded, and the spore-containing precipitate was rinsed twice with sterile water to remove any residual medium. A further centrifugation step was performed, and the supernatant discarded, leaving a pink precipitate which comprised the harvested conidia. Finally, 10 mL of sterile water was added to the centrifuge tube and the conidia were resuspended via pipetting, resulting in a highly concentrated conidial suspension. After shake flask cultures, the conidia germinate into hyphae were sent to Major.bio(Shanghai, China) for RNA-seq analysis. Analyzed data visualization was carried out in R with the ggplot2 package.

### 2.3. Domain Architecture of CfPmt4 and Phylogenetic Tree

The domains of CfPmt4 were predicted by SMART (http://smart.embl-heidelberg.de/smart/set_mode.cgi?NORMAL=1, accessed on 18 January 2024). The Pmt4 proteins in *Magnaporthe oryzae* (XP003713520), *Madurella mycetomatis* (KXX79740), *Fusarium oxysporum* (KAG7421719), *Tolypocladium capitatum* (PNY23596), *C. fructicola* (KAF4491891), *C. viniferum* (KAF4929753), *Alternaria arborescens* (XP028508322), and *Saccharomyces cerevisiae* (AJP39822) were obtained from the NCBI database. The MEGA 7.0 program was used to construct the phylogenetic analysis (http://www.megasoftware.net/, accessed on 20 January 2024).

### 2.4. Gene Replacement and Complementation

Using wild-type CFLH16 genomic DNA as template, PCR amplification was performed using primers *CfPMT4*-1F/*CfPMT4*-2R and *CfPMT4*-3F/*CfPMT4*-4R, and the sequences of the upstream and downstream coding regions of the *CfPMT4* gene were about 1kb each, and the primers used in this study are listed in Table 1. Hygromycin resistance cassette (HPH) was amplified with Hyg-F/Hyg-R as the primer. Finally, using the above three fragments as templates, the primer *CfPMT4*-1F/*CfPMT4*-4R was added for fusion PCR to obtain the knockout fragment of the *CfPMT4* gene, which was then transformed into protoplasts of CFLH16, as was carried out by Li et al. [9].

The PEG-mediated protoplast transformation method was utilized to generate a gene knockout mutant by introducing the *CfPMT4* gene knockout fragment into wild-type CFLH16 protoplasts, followed by screening and cultivation on a hygromycin-containing TB3 culture medium. Subsequent verification through agarose gel electrophoresis involved the amplification of transformant DNA using inner primers (*CfPMT4*-7F/*CfPMT4*-8R) and outer primers (*CfPMT4*-5F/H855R) specific to the *CfPMT4* gene. The mutant strain was identified when the PCR failed to amplify strips of correct sizes with inner primers, but successfully amplified them with outer primers.

The complement fragment of the *CfPMT4* gene was obtained by PCR amplification using primer *CfPMT4*-9F/*CfPMT4*-10R, which was transferred into yeast receptor cell XK-125 together with the pYF11(bleomycin resistance) vector, as was previously described. Then, the plasmid with correct sequencing was transformed into the protoplast of the Δ*Cfpmt4* to obtain the complementary strains.

### 2.5. Pathogenicity Assays

Twenty microliters of conidial suspensions of WT, Δ*Cfpmt4*, and Δ*Cfpmt4*/*CfPMT4* containing 1 × 10^6^ spores/mL were inoculated onto the peripheries of healthy or wounded young tea-oil leaves and placed on high-humidity plates for a duration of 4 days. Subsequently, the resulting lesions were observed and analyzed with ImageJ (V1.8.0). To further investigate the specificity of the tea-oil tree in relation to the low-pathogenicity of mutants, multiple 8 mm diameter holes were initially created in the epidermises of mature apples. Mycelial plugs measuring 3 mm × 3 mm were then introduced into the holes. Following an incubation period of 5 to 7 days, the lesions (calculated as the total area minus the area of the hole) were evaluated by ImageJ (V1.8.0).

### 2.6. Growth, Conidiation, and Appressoria Formation Assays

The strains were cultivated on CM and minimal medium (MM) agar plates at a temperature of 28 °C under conditions of darkness for a duration of 3 days. Subsequently, the diameters of the colonies were measured and subjected to statistical analysis. In order to assess conidiation, the strains were cultivated in 50 mL of liquid shaking CM for a period of 2 days, after which they were quantified using a hemocytometer under a microscope. For the purpose of evaluating appressoria formation, the collected conidia were resuspended to a concentration of 1 × 10^5^ spores/mL and then inoculated onto hydrophobic artificial surfaces to induce appressorium formation. The experiments were repeated three times with three replicates each time.

### 2.7. Stress Response Assays

The strains were cultivated on CM and CM with cell wall integrity stress (400 µg/mL CR) or ER stress (2.0 μg/mL TM and 5.0 mmol/mL DTT). Following a 3-day incubation period, the diameters of the colonies were measured, and the inhibition rates were subjected to statistical analysis.

### 2.8. Glycogen Metabolism

A total of 10 μL drops of conidial suspensions of each strain were added to a hydrophobic slide and cultured in the dark at 28 °C. Iodine staining (120 mg/mL KI + 20 mg/mL I_2_) was performed and the glycogen staining in conidium and appressorium was observed under microscope [10].

### 2.9. Statistical Analysis

The statistical data were presented as mean ± standard deviation (SD) and subjected to a one-way analysis of variance (ANOVA) and Duncan’s new multiple-range test, with a significance level of *p* < 0.01.

## 3. Results

### 3.1. GO Enrichment Analysis of Differentially Expressed Genes (DEGs)

**Figure 1:**
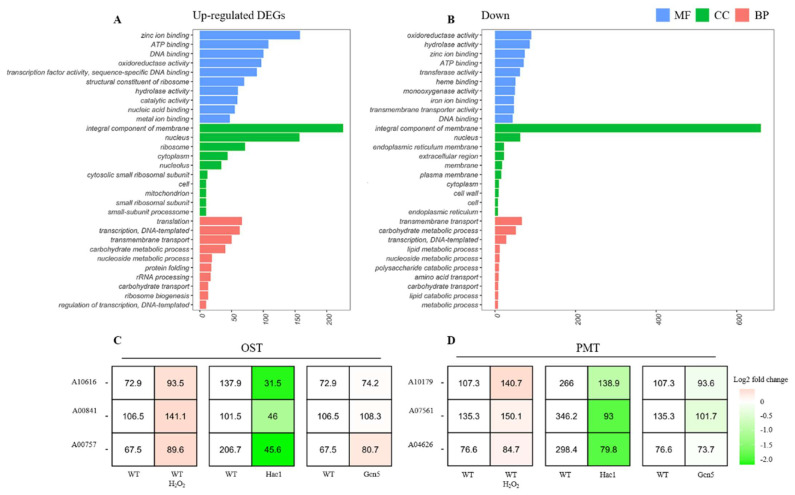
GO enrichment analysis of differentially expressed genes (DEGs) and gene expression of *CfOSTs* and *CfPMTs*. (**A**) and (**B**) are DEGs being up−and down−regulated in wild−type strain relative to Δ*Cfhac1*; (**C**) and (**D**) are the expression patterns of identified *OST/PMT* genes in *C. fructicola*. The heatmap encodes gene responses to stress, with expression changes represented as log2−fold against a control, with up−regulation in red and down−regulation in green.

In an endeavor to comprehend the varying roles of certain enzymes in diverse biological processes and cellular functions, a comparative Gene Ontology (GO) enrichment analysis was conducted between the wild-type strain and the CfHac1 mutant (Figure 1A,B). This analysis highlighted the enriched up- and down-regulated genes within the Hac1 mutant and underscored both commonalities and differences between the gene sets. We observed common molecular functions, such as ‘zinc ion binding’, ‘ATP binding’, and ‘DNA binding’ among others, but also identified unique functions specific to each gene set. We noted ‘RNA polymerase II transcription factor activity, sequence-specific DNA binding’, and ‘structural constituent of ribosome’ exclusively in the up-regulated genes, indicating their context-specific roles. The analysis of cellular components demonstrated that both sets are predominantly composed of membrane proteins. However, we also observed a broader cellular distribution of genes within the up-regulated set compared to the down-regulated set, which showed a stronger association with ‘endoplasmic reticulum membrane’ and ‘extracellular region’. The examination of the biological processes revealed that both gene sets participated in processes like ‘transcription, DNA-templated’, ‘transmembrane transport’, and ‘carbohydrate metabolic process’, underlining their shared biological roles, yet their differential distribution suggests they operate in different biological contexts. Importantly, the GO analysis suggested an increased presence of ‘endoplasmic reticulum membrane’-related genes in the Δ*Cfhac1* mutant, pointing towards a potential shift in cellular function upon Δ*Cfhac1* mutation. Moreover, the unique enrichment of ‘protein glycosylation’-related genes, linked with oligosaccharyltransferase and protein *O*-Mannosyltransferases (OST/PMT) function, suggested a possible interaction between *HAC1* and *OST/PMT* genes. Consequently, we identified all *OST/PMT* genes in *C. fructicola* and analyzed their expression levels. To elucidate novel glycosylation-related genes that may be important for *C. fructicola*’s virulence, we took an unbiased transcriptomic approach comparing wild-type and the ∆*Cfhac1* mutant. Notably, all genes were induced by dithiothreitol and down-regulated in the Δ*Cfhac1* mutant, yet only two OST genes (A10616 and A00841) and one PMT gene (A04626) were unaffected in the Δ*Cfgcn5* (Figure 1C,D). This specificity of Hac1 and the OST/PMTs supports the investigation of protein *O*-mannosyltransferases (PMTs), less-explored enzymes in ER stress, to further our understanding of the unfolded protein response (UPR), crucial for maintaining cellular homeostasis. Unraveling the interplay between PMTs and UPR, regulated by *HAC1*, offers unique insights into cellular responses and therapeutic potential for numerous diseases. Following this, we classified A04626 as *CfPMT4* through further homolog analysis.

### 3.2. Phylogenetic Analysis of CfPmt4 in C. fructicola

**Figure 2:**
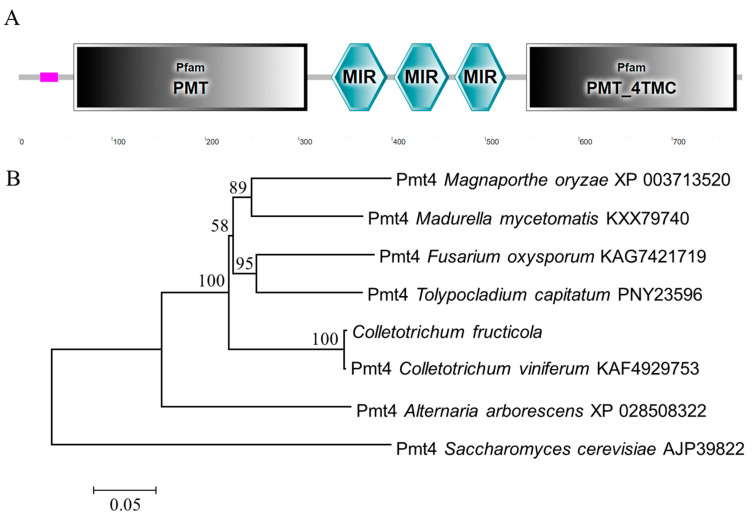
CfPmt4 domain prediction and phylogenetic analysis. (**A**) Domain prediction of CfPmt4 protein using SMART website; scale represents number of amino acids. (**B**) Phylogenetic tree of CfPmt4 and its fungal homologs constructed using MEGA 7.0 software with the neighbor-joining method. Pmt4 in Saccharomyces cerevisiae (AJP39822) was used as an outgroup.

The *CfPMT4* gene (GenBank accession number: KAF4491891) was found to have a total length of 2451 base pairs, encoding a protein consisting of 775 amino acids. The analysis of protein domains revealed the presence of a PMT domain, three MIR domains, and a PMT_4TMC domain within the CfPmt4 (Figure 2A). The phylogenetic analysis revealed a significant level of homology between the CfPmt4 protein of *C. fructicola* and the protein of *Colletotrichum viniferum*, while displaying a comparatively lower level of homology with the protein of *S. cerevisiae* (Figure 2B).

### 3.3. Targeted Deletion of the CfPMT4 Gene in C. fructicola

**Figure 3:**
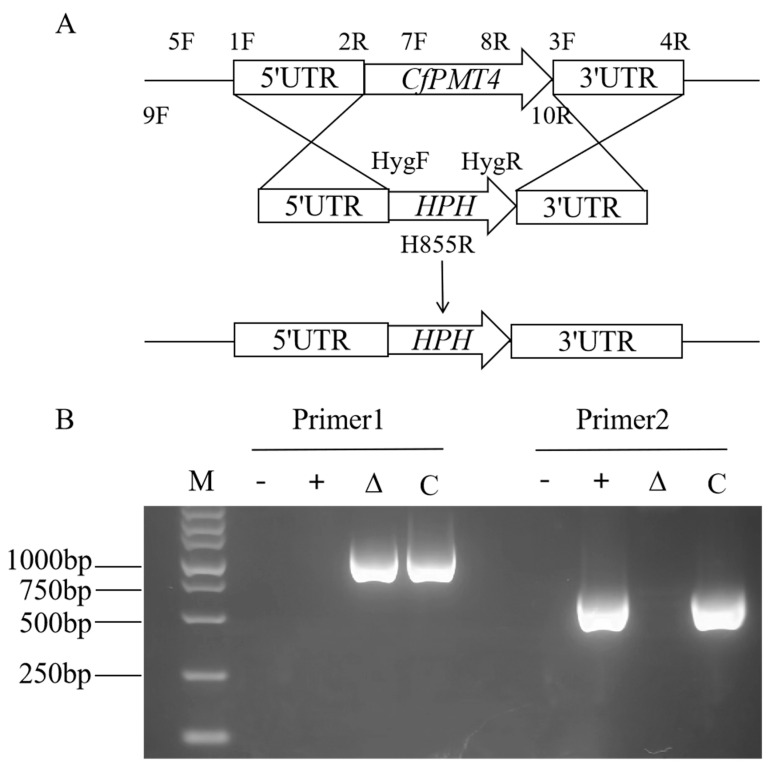
Acquisition of *CfPMT4* gene knockout mutant. (**A**) Template of gene knockout strategy; (**B**) verification of mutants; primer pair 1: CfPMT4-5F/H855R; primer pair 2: CfPMT4-7F/CfPMT4-8R; M: marker with a molecular weight of 5000 bp; -: sterile water negative control; +: wild-type; Δ: mutant; C: complementary strains.

In order to study the biological function of CfPmt4, the *CfPMT4* gene was knocked out by homologous recombination, and the strategy is shown in Figure 3A. The construction of the mutant strains and complemented strains was carried out following the methodology outlined in Ref. [6]. The results of this experiment are provided in Figure 3B.

### 3.4. CfPmt4 Regulates Pathogenicity of C. fructicola

**Figure 4:**
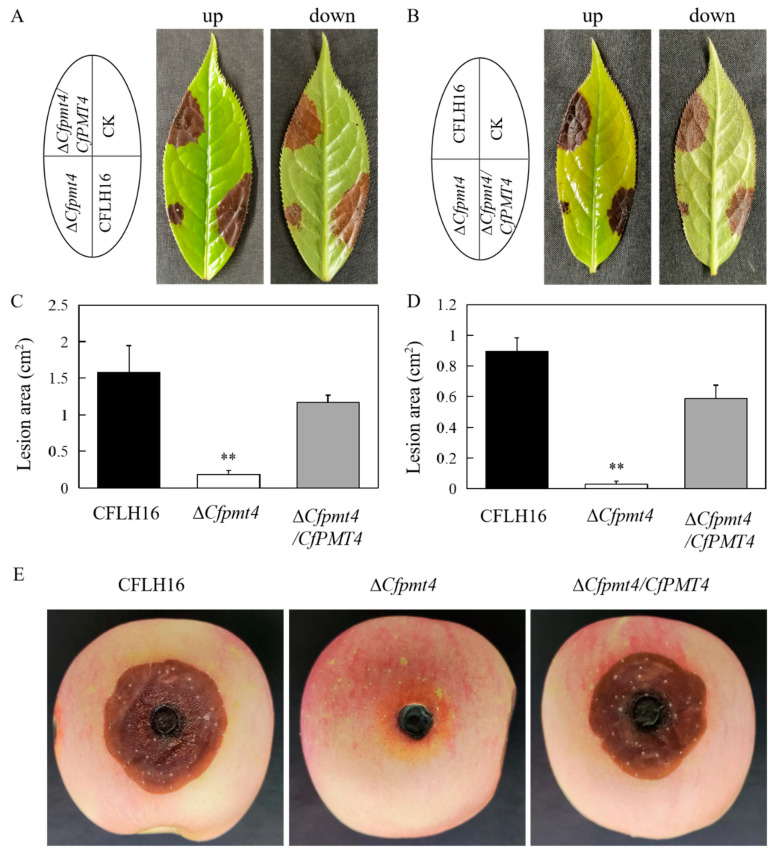
Pathogenicity of mutant Δ*Cfpmt4*. (**A**,**B**) Pathogenic conditions of wild-type, mutant, and complementary strains on injured and uninjured *Camellia* leaves, respectively; (**C**,**D**) statistical analysis of the diameter of the spots produced by three strains on injured and uninjured *Camellia* leaves; (**E**) pathogenic effects of wild-type, mutant, and complementary strains on apple; CK: sterile medium; ** means very significant difference (*p* < 0.01).

Whether CfPmt4 affects the pathogenicity is the focus of our study. The results of the pathogenicity test showed that the lesion area in mutant strain Δ*Cfpmt4* was significantly less than the large and typical lesions caused by wild-type and Δ*Cfpmt4*/*CfPMT4* (Figure 4A–D). In addition, in order to ascertain the tea-oil tree’s specificity in relation to the low-pathogenicity of Δ*Cfpmt4*, mycelial plugs were introduced to wounded apples. Consequently, Δ*Cfpmt4* exhibited smaller lesions, whereas wild-type and Δ*Cfpmt4*/*CfPMT4* displayed typical lesions (Figure 4E). These findings collectively suggest that CfPmt4 plays a vital role in pathogenicity. 

### 3.5. CfPmt4 Regulates Vegetative Growth

**Figure 5:**
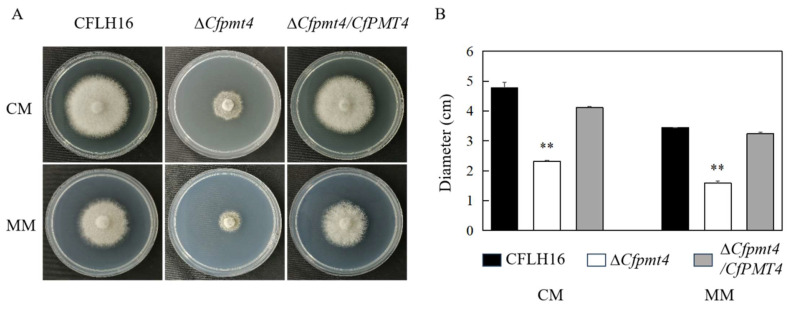
Vegetative growth of mutant Δ*Cfpmt4*. (**A**): The growth of wild-type, mutant, and complementary strains on CM and MM medium; (**B**): statistical analysis of colony diameter of three strains; ** means very significant difference (*p* < 0.01).

In order to examine the involvement of the *CfPMT4* gene in the vegetative growth of *C. fructicola*, an investigation was conducted on the growth of Δ*Cfpmt4* on two distinct culture mediums, CM and MM (Figure 5A). The results obtained demonstrated significantly smaller diameters of Δ*Cfpmt4* in comparison to the wild-type and Δ*Cfpmt4*/*CfPMT4* on both culture mediums (Figure 5B). These findings revealed that *CfPMT4* is involved in vegetative growth in *C. fructicola*.

### 3.6. CfPmt4 Regulates Conidiation and Appressorium Formation

**Figure 6:**
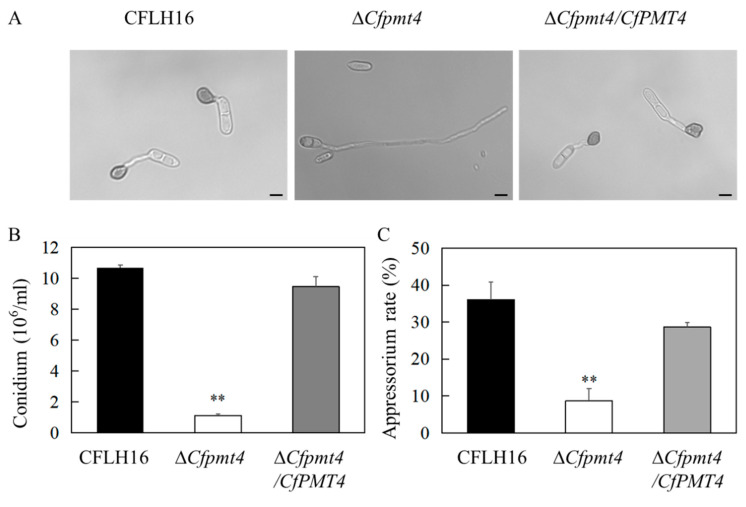
Sporulation determination of mutant ΔCfpmt4. (**A**) The appressorium formation of wild- type, mutant and complementary strains after the same time of moisturizing culture; (**B**) statistical analysis of spore production of wild-type, mutant, and complementary strains; (**C**) statistical analysis of appressoria formation rates of three strains; ** indicates a statistically significant difference (*p* < 0.01); scale bar: 5 μm.

Conidia are asexual spores and play a particularly crucial role in fungal dissemination. Appressoria play a vital role in the infection process of the phytopathogenic fungus. In order to induce sporulation, the strains were introduced into the PDB medium. Our findings indicate a noteworthy decrease in sporulation within the Δ*Cfpmt4* mutant when compared to the wild-type (WT) and complemented strains (Figure 6B). The appressorium serves as the primary infection structure of the fungal pathogen to infect plants. Subsequent examination revealed that the mutant strain Δ*Cfpmt4* exhibited a notably reduced rate of appressorium formation, approximately 8%, in comparison to the wild-type and complementary strains (Figure 6A,C). These results provide evidence for the critical role of the *CfPMT4* gene in the conidiation and appressorium formation processes of *C. fructicola*.

### 3.7. CfPmt4 Plays Crucial Roles in Cell Wall Integrity

**Figure 7:**
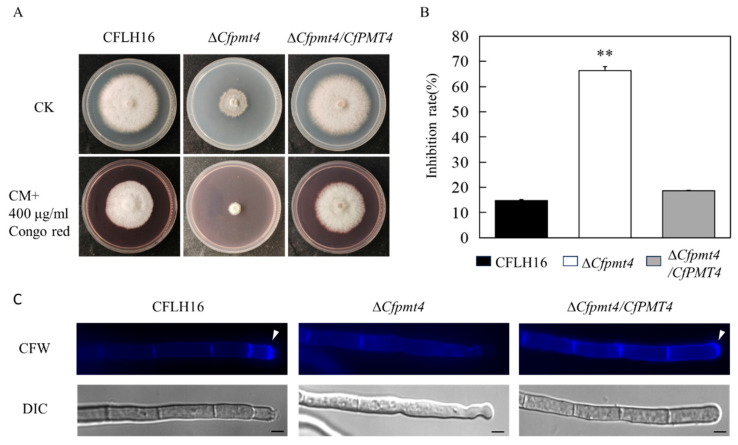
Cell wall integrity of the mutant Δ*Cfpmt4*. (**A**) The growth of wild-type, mutant, and complementary strains under 400 µg/mL CR stress; CK, control check; (**B**) statistical analysis of colony diameter of three strains; (**C**) the mycelia of the strains were stained with 10 mg/mL of CFW for 5 min in the dark before being photographed; arrows indicate the stained hyphal tips. The experiment was repeated three times with triplicates, which showed the same results. DIC, differential interference contrast image; ** means extremely significant difference (*p* < 0.01); scale bar: 5 μm.

Pathogenic fungi are confronted with different cell wall stressors in natural conditions. Thus, we tested the roles of CfPmt4 in the responses to cell wall stress. The results showed that Δ*Cfpmt4* exhibited higher inhibition rates (>60%) in Congo red than the wild-type and Δ*Cfpmt4*/*CfPMT4* (<20%) (Figure 7A,B). These findings revealed that *CfPMT4* is essential for the responses of *C. fructicola* to cell wall stress. CFW staining was utilized to evaluate the localization of chitin at the apex of Δ*Cfpmt4* hyphae, indicating no aggregation of chitin at the hyphal tip in the Δ*Cfpmt4* strain. Conversely, in the wild-type and Δ*Cfpmt4*/*CfPMT4* strains, chitin predominantly accumulated at the hyphal tip (Figure 7C). These findings suggest that the *CfPMT4* gene was involved in modulating the distribution of chitin within hyphae.

### 3.8. CfPmt4 Participates in the Responses to Endoplasmic Reticulum Stress

**Figure 8:**
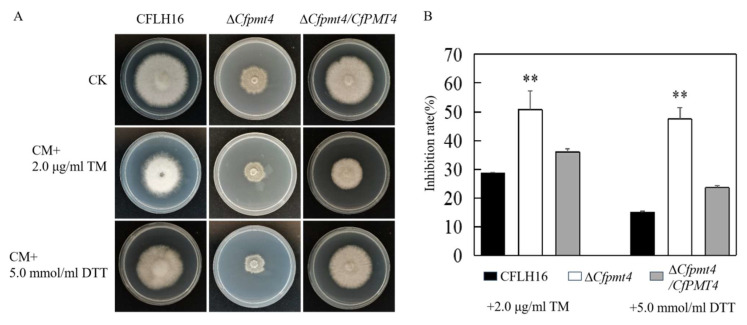
Response of the mutant Δ*Cfpmt4* to endoplasmic reticulum stress. (**A**): Growth of wild-type, mutant, and complementary strains under 2.0 µg/mL TM and 5.0 mmol/mL DTT stress, respectively; (**B**): statistical analysis of colony diameter of three strains; ** means very significant difference (*p* < 0.01).

According to recent studies, pathogens also experience ER stress derived from the host during infection [11]. So, we mimicked plant ER stress using TM and DTT. The findings of our study indicated that when treated with 2.0 µg/mL TM, Δ*Cfpmt4* showed an inhibition rate of 50.7%, in contrast to that of 28.7% and 35.9% in WT and Δ*Cfpmt4*/*CfPMT4*, respectively. A similar result was observed when treated with 5.0 mM DTT, Δ*Cfpmt4* showed an inhibition rate of 47.5%, in contrast to that of 15.0% and 23.5% in WT and Δ*Cfpmt4*/*CfPMT4*, respectively. Furthermore, the analysis of inhibition rates revealed a significant reduction in the resistance of the mutant strain to TM and DTT (Figure 8A,B). The results of this study indicated that the *CfPMT4* gene of *C. fructicola* participates in the regulation of the response to ER stress.

### 3.9. CfPmt4 Is Essential for Glycogen Metabolism

**Figure 9:**
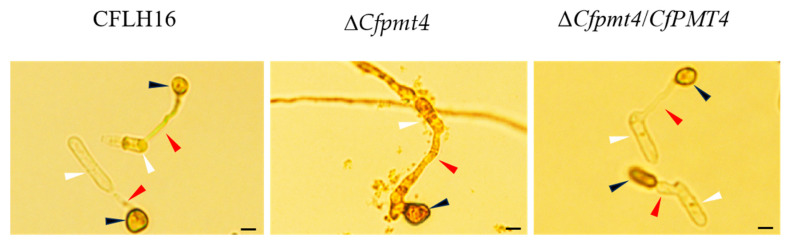
Observation of iodine staining of Δ*Cfpmt4*. Red arrows indicate germ tubes; black arrows indicate appressoria; white arrows indicate conidia; scale bar: 5 μm.

The rapid degradation of lipid droplets and glycogen plays a crucial role in the generation of turgor pressure during the development of appressoria. In our study we found that the Δ*Cfpmt4* mutants showed a retardation in the translocation of glycogen to appressoria during appressorium formation, in comparison with wild-type and Δ*Cfpmt4*/*CfPMT4* (Figure 9), indicating that CfPmt4 is involved in glycogen metabolism in *C. fructicola*.

## 4. Discussion

The conservation of Pmts proteins in fungi is responsible for the initiation of the first mannose residues during glycosylation. This process has a significant impact on the structure and function of both secreted and membrane proteins, as well as regulating the various aspects of fungal growth and development, including sporulation, appressorium formation, cell wall integrity, stress response, and pathogenicity. In *Saccharomyces cerevisiae*, protein *O*-Mannosyltransferases (PMTs) family members are divided into three subfamilies: Pmt1p, Pmt2p, and Pmt4p [12]. Studies have shown that fungal o-mannosylation has many roles, including cell morphology, growth, cell wall integrity, protein stability, sorting, and localization [13]. In human pathogens *Candida albicans* and *Cryptococcus neoformans*, Pmt4 affects cell wall integrity and pathogenicity [14,15]. The *PMT4* gene of *Aspergillus fumigatus* plays a crucial role in the modulation of mycelial growth, sporulation quantity, and polarity [16]. The Pmt4 of *Ustilago maydis* is essential in its pathogenic process, and its absence leads to a significant reduction in the rate of appressorium formation [17]. Th deletion of *PMT4* gene in *Magnaporthe oryzae* resulted in slower growth and development, reduced cell wall integrity and virulence [18]. Drawing from the evidence, it is posited that Pmt4 assumes a pivotal function in the proliferation and pathogenicity of fungi. The results of our experiment indicate that the deletion of the *CfPMT4* gene resulted in a significantly smaller lesion area compared to the wild-type, suggesting that CfPmt4 plays a regulatory role in the pathogenic process. The findings are in line with the manifestation observed in most filamentous fungi, including *Botrytis cinerea*, *Fusarium oxysporum*, and *Beauveria bassiana* [19,20,21]. Conidia play an important role in the disease cycle of *C. fructicola*. Compared with the WT strain, the Δ*Cfpmt4* mutants did not differ in conidial morphology but had significantly reduced sporulation ability. In *M. oryzae*, the deletion of the *PMT4* gene significantly reduced sporulation and malformed conidia [22]. There is speculation that the *PMT4* gene may serve varying functions across distinct species.

The formation of the appressorium via conidial germination is a critical process in disease development, as it influences the molecular interactions between pathogenic fungi and host plants, thereby facilitating fungal invasion [23]. This study revealed that CfPmt4 was involved in regulating the formation of appressoria and subsequently influences the pathogenic process.

The role of high turgor pressure is crucial in the development of invasive structures that penetrate the surface of the host. According to contemporary research, the significant turgor pressure produced during the process of appressorium formation is intricately linked to the breakdown of glycogen and lipids into glycerol [24]. The results of the iodine staining experiment indicate that the absence of the *CfPMT4* gene led to hindrance in glycogen transfer and metabolism, which may be the main reason for the reduction in the virulence of Δ*Cfpmt4*.

When the endoplasmic reticulum (ER) undergoes excessive stress, it may undergo misfolding, prompting cells to initiate the unfolded protein response (UPR) to restore normal function. The UPR pathway is a conserved eukaryotic signaling mechanism that detects misfolded proteins within the ER and subsequently triggers downstream transcription factors. This activation induces the expression of genes that facilitate the protein folding, secretion, and degradation of misfolded proteins, ultimately alleviating ER stress. O-Mannosylation is a type of protein glycosylation initiated in the endoplasmic reticulum by the protein *O*-mannosyltransferase (PMT) family. The findings of this investigation indicate that the lack of the *PMT4* gene in *C. fructicola* led to notable inhibition under the influence of TM and DTT stress. This suggests that CfPmt4 played a significant role in regulating the response to endoplasmic reticulum stress. Our study provides evidence of the participation of CfPmt4 in the modulation of responses to endoplasmic reticulum stresses, which could potentially contribute to the regulatory role in pathogenicity.

In conclusion, it is postulated that the regulatory role of CfPmt4 extends to the pathogenicity of *C. fructicola*, encompassing the regulation of conidia production, appressoria formation, cell wall integrity, endoplasmic reticulum folding protein response, and glycogen metabolism. Furthermore, the Δ*Cfpmt4* mutant exhibited reduced growth in response to CR stress, suggesting a compromised cell wall integrity. Moreover, it is imperative to investigate the protein structure of CfPmt4 and explore potential inhibitors that could impede its function, ultimately resulting in a reduction in virulence.

## Figures and Tables

**Table 1:** Primers used in this study.

Primer	Sequence	Target Region
*CfPMT4*-1F	CCTTGTCTGAGGTTCGTCAA	amplify *CfPMT4* 5′ flank sequence
*CfPMT4*-2R	GTTGGGAATCGGAAATGGAC	amplify *CfPMT4* 5′ flank sequence
*CfPMT4*-3F	GCGCCGTGAGGTTGAAGTCA	amplify *CfPMT4* 3′ flank sequence
*CfPMT4*-4R	TTCCAGTGCTCCTTGATCAG	amplify *CfPMT4* 3′ flank sequence
*CfPMT4*-5F	CGACCGAGAGTTTACGTACA	validation of *CfPMT4* gene deletion
H855R	GCTGATCTGACCAGTTGC	validation of *CfPMT4* gene deletion
*CfPMT4*-7F	ACGACGGACACTTCCACTTC	amplify *CfPMT4* gene sequence
*CfPMT4*-8R	GGCCAACATGACGTTATCGC	amplify *CfPMT4* gene sequence
*CfPMT4*-9F	CGAGGGCATTCAGAAAAGAC	amplify complemented sequence
*CfPMT4*-10R	TTTGGCGAAGTGCAGGTCGT	amplify complemented sequence
Hyg-F	GGCTTGGCTCCAGCTAGTGGAGGT	amplify *HPH* sequence
Hyg-R	CTCTATTCCTTTGCCCTCG	amplify *HPH* sequence

## Data Availability

All data supporting the findings of this study are available within the paper.
